# Isolation and Characterization of Human Mesenchymal Stem Cells Derived from Human Umbilical Cord Wharton’s Jelly and Amniotic Membrane

**Published:** 2013-08-01

**Authors:** T. Pirjali, N. Azarpira, M. Ayatollahi, M. H. Aghdaie, B. Geramizadeh, T. Talai

**Affiliations:** 1 *Transplant Research Center, Shiraz University of Medical Science, Shiraz, Iran*; 2 *Islamic Azad University, Fars Science and Research Branch, Shiraz, Iran*; 3 *Shiraz Institute of Stem Cell and Regenerative Medicine, Shiraz University of Medical Science, Shiraz, Iran *; 4 *Department of Anatomy, Shiraz University of Medical Science, Shiraz, Iran*

**Keywords:** Mesenchymal Stromal cells, Amnion, Umbilical cord

## Abstract

Background: Mesenchymal stem cells (MSCs) have a capacity for self-renewal and multi-potential differentiations. These cells are considered powerful sources for cell therapy in regenerative medicine and tissue engineering. The cells can be isolated from various tissues; however, harvesting from human umbilical cord and amniotic membrane is easy and accessible source.

Objective: To isolate and characterize the MSCs derived from human umbilical cord Wharton’s jelly (WJ-MSC) and amniotic membrane (AM-MSC) with regard to their morphology, immunophenotype and mesodermal differentiation potential in order to obtain an alternative source of MSC for therapeutic clinical applications.

Methods: Fetal membranes and umbilical cords (n=3) were retrieved from healthy full-term women by elective cesarean delivery. Amniotic membrane and umbilical cord were separately minced and cultured in DMEM supplemented with 10% FBS. After reaching 80% of confluency, the umbilical cord WJ-MSC and AM-MSC were characterized by expression of cell surface markers with flowcytometry, stem cell gene expression with adipogenic/osteogenic potential.

Results: Both WJ-MSC and AM-MSC were spindle-shaped cells, expressed MSC surface markers in flowcytometry and stem cell transcriptional factors (OCT4 and NANOG). After induction, the cells differentiated into adipogenic and osteogenic lineages.

Conclusion: MSC were successfully generated from umbilical cord WJ-MSC and AM-MSC with similar mesenchymal markers and properties.

## INTRODUCTION

Mesenchymal stem cells (MSCs) are cells with high *in vitro *self-renewal capacity and ability to differentiate into multiple mesoderm, ectoderm and endoderm lineages. They also possess anti-inflammatory and immuno-modulatory effects and are considered as a promising tool for cell-based therapeutic strategies in regenerative medicine [1-3].

MSCs were originally isolated from bone marrow (BM), but for their clinical application, several limitations exist. BM aspiration is an invasive procedure; the cell self-renewal ability of BM-MSCs is markedly decreased with increasing donor age. On the other hand, in regenerative medicine, clinically relevant cell numbers with appropriate quality must be achieved within a very limited time [2, 3].

Therefore, fetal tissue is considered a good alternative source with high-yield for stem cell recovery. It is usually discarded without any ethical conflict [4, 5].

Eight days after fertilization, the human blastocyst is embedded in the endometrium. It is composed of outer and inner cell mass. The outer cells (trophoblast) differentiate into two layers (Cytotrophoblast and syncytiotrophoblast) and migrate into the endometrial stroma. The inner cell mass or embryoblast differentiates into two layers, the hypoblast and epiblast.

The amniotic cavity appears from epiblast. The three germ layers, endoderm, mesoderm, and ectoderm were developed from epiblast. Amniotic membrane has two cell types: amnion epithelial cells derive from embryonic ectoderm and amnion mesenchymal cells from embryonic mesoderm [6].

At the first time, In ‘t Anker, *et al*, showed that amniotic membrane contains MSCs with osteogenic and adipogenic differentiation potential [7]. Wharton’s jelly is the primitive connective tissue of the umbilical cord presents between the amniotic epithelium and the umbilical vessels. The major role of Wharton’s jelly is to prevent compression and torsion of the umbilical vessels in fetus [2, 8]. Wharton’s jelly also have primitive MSCs, trapped in the connective tissue matrix as the hematopoietic and mesenchymal cells migrate through the early umbilical cord to the placenta during embryogenesis [2, 8].

We performed the present study to isolate and characterize the MSCs derived from human umbilical cord Wharton’s jelly (WJ-MSC) and amniotic membrane (AM-MSC) with regard to their morphology, immunophenotype and mesodermal differentiation potential to obtain an alternative source of MSC for therapeutic clinical applications.

## MATERIAL AND METHODS

Tissue collection and processing 

Written informed consent was obtained from each patient prior to collection; ethics approval was obtained from the Ethics Committee of Shiraz University of Medical Sciences.

Fetal membranes and umbilical cords (n=3) were retrieved from healthy full-term women who underwent elective cesarean section. The amnion was peeled from the chorion and rinsed in phosphate-buffered saline (PBS) to remove blood. The tissue pieces were transferred to 10 cm^2 ^plates containing DMEM supplemented with 10% FBS+penicillin 100 U/mL, streptomycin 100 µg/mL, and incubated at 37 °C in a humidified atmosphere containing 5% CO_2._

The cord was washed, blood vessels removed and tissue cut into pieces (1 mm2) and cultured in culture plates. The culture expansion of small pieces of tissue is known as the “explants method.” The explants were left undisturbed for one week to allow the migration of cells from the margins of explants. After reaching 70% to 80% confluency, adherent cells was harvested with trypsinization by 0.05% trypsin-EDTA (Gibco, Germany); a single cell suspension was used for subsequent experiments.

Immunophenotyping by Flow Cytometry 

To phenotype cell-surface antigens, the fourth-passage cells were stained with monoclonal antibodies specific for the following proteins CD90-FITC, CD45-FITC, CD133-PE, CD44- FITC, CD-34- FITC, CD133-PE and CD105-FITC (BioLegend, San Diego, CA, USA). Cells were also treated with appropriate isotype control antibodies. Stained cells were re-suspended in PBS, analyzed using FACS Calibur flowcytometer (Becton Dickinson, NJ, USA). For each sample, at least 10,000 events were recorded.

Mesodermal lineage differentiation assay 

Commercial adipocytes and osteocytes differentiation media (Bonyakhteh, Iran) was used for *in vitro* differentiation of these cells. After differentiation, the adipocytes and osteocytes were fixed and stained with Oil Red O and Alizarin Red, respectively.

RT-PCR analysis 

Total RNA was extracted using RNX extraction kit (Cinagene, Iran). Following DNase treatment, 1 µg total RNA was reverse-transcribed into cDNA using random primers, Moloney murine leukemia virus (MMLV) reverse transcriptase and RNase inhibitor (Cinagene, Iran).

The primer pairs for amplification of OCT (bp of 161) and NANOG (bp of 111) were:

OCT-F: 5’-CAGTGCCCGAAACCCACAC-3’

OCT-R: 5’-GGAGACCCAGCAGCCTCAAA-3’

NANOG-F: 5’-CAGAAGGCCTCAGCACCTAC-3’

NANOG-R: 5’-ATTGTTCCAGGTCTGGTTGC-3’

Amplification reactions were performed on the following conditions: denaturation at 95 °C for 10 min followed by 40 cycles of denaturation at 95 °C for 30 s, annealing at 65 °C for 40 s, and elongation at 72 °C for 5 min. The PCR products were visualized following electrophoresis on 3% (w/v) agarose gel containing Safe stain (Cinagene, Iran).

## RESULTS

Morphological features of AM-MSC 

**Figure 1:**
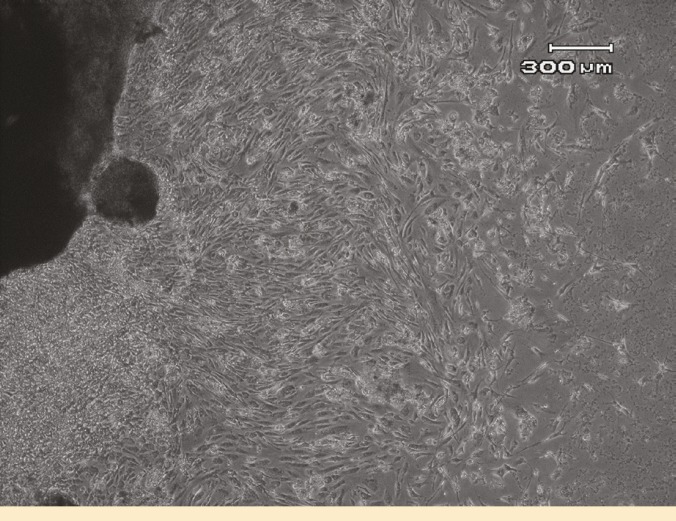
Heterogeneous population of amnion-derived MSCs with fibroblastic and epithelioid morphology at passage 0.

**Figure 2:**
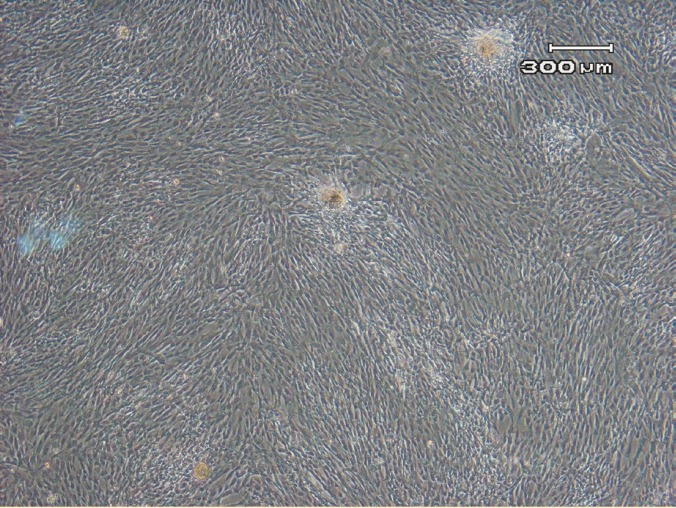
Homogenous population of fibroblast-like MSCs at passage 3.

The initial growth of AM-MSC at passage 0 (P0) consisted of adherent cells with heterogenous population; one of them was spindle-shaped fibroblast-like cells and another one had epithelial-like morphology (Fig 1). The cells became confluent after a mean of 14 days. After trypsinization and subculture, the epithelioid cells disappeared from the culture. The WJ-MSCs formed a homogenous monolayer of adherent, spindle-shaped fibroblast-like cells in primary culture (P0) (Fig 2).

**Figure 3:**
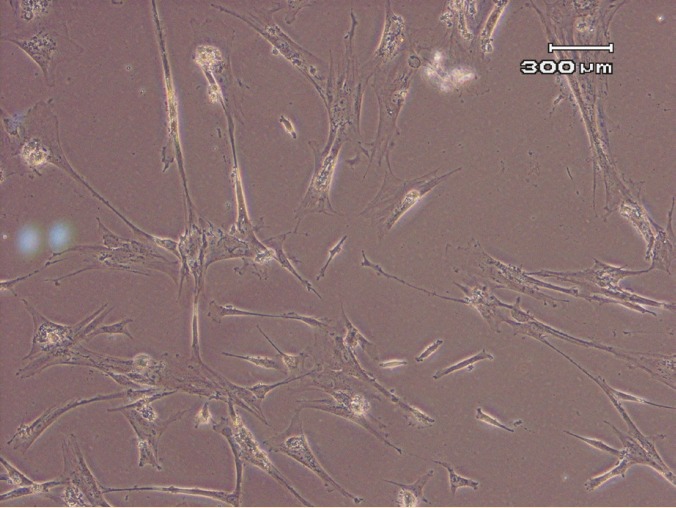
MSCs at late passage with large and less adherent. Photomicrographs taken by phase contrast microscope (×100 and ×400).

The MSCs were cultured and expanded until the 9^th^ passage (P10). At early passages, the cells proliferated rapidly with small-sized spindle-shaped cells. But the features gradually changed at later passages (P9 onwards), where both AM-MSC and WJ-MSCs showed morphological changes; they appeared unhealthy, became larger and eventually died (Fig 3).

Characterization of CP-MSCs and WJ-MSCs 

The results of RT-PCR analyses showed that both AM-MSC and WJ-MSCs expressed pluripotent stem cell markers of Oct4 and Nanog.

**Figure 4:**
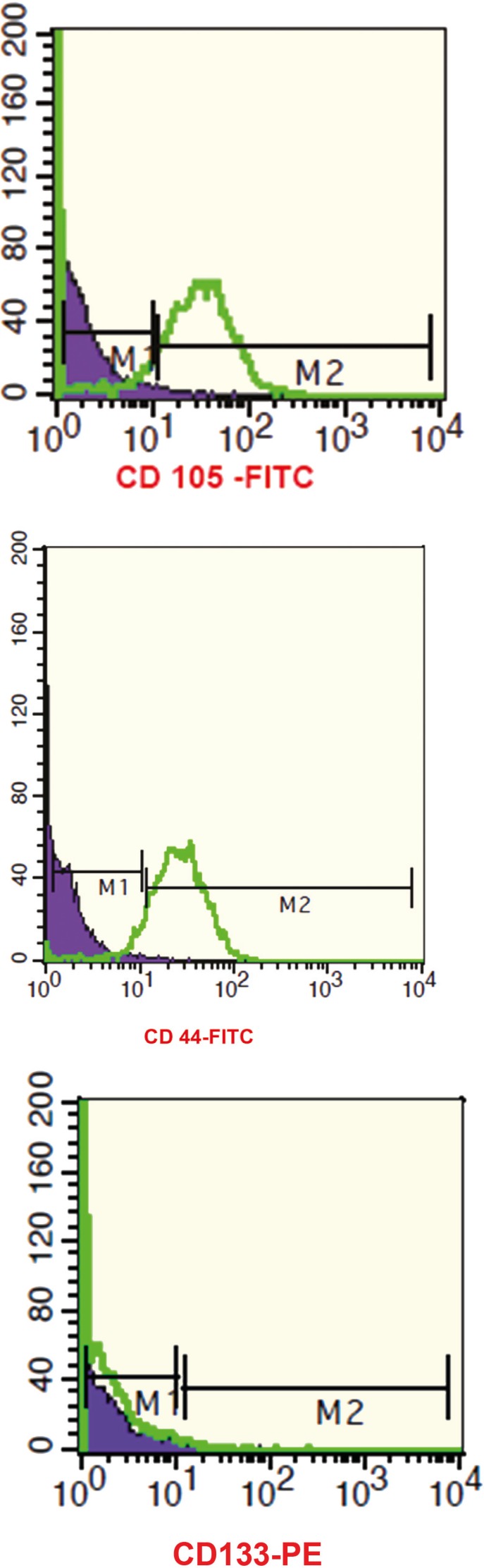
Immunophenotyping of MSCs by flowcytometry. In each case, blue histograms indicate isotype matched IgG controls.

The immunophenotypes of AM-MSC and WJ-MSCs were analyzed by flowcytometry. More than 90% of AM-MSC and WJ-MSCs were positive for the MSC markers, CD105 and CD90, and were negative for hematopoietic and endothelial cell markers, CD45, CD34 and CD133 (Fig 4).

Differentiation of AM-MSC and WJ-MSCs to mesodermal-lineage cells 

**Figure 5:**
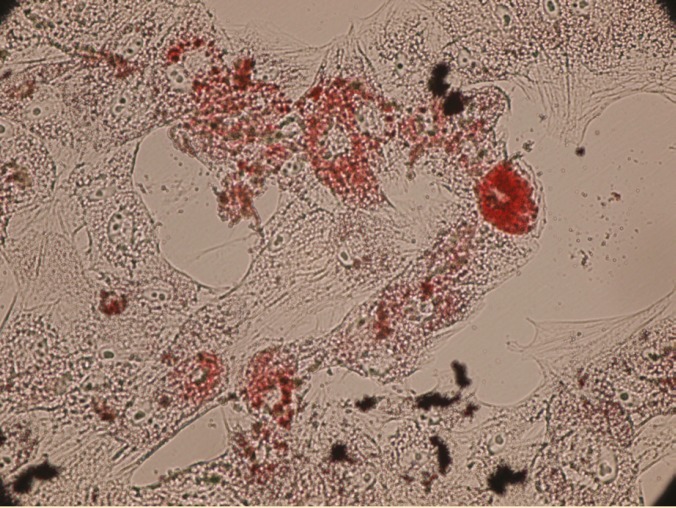
Lipid vacuoles stained with Oil Red O (×100)

**Figure 6:**
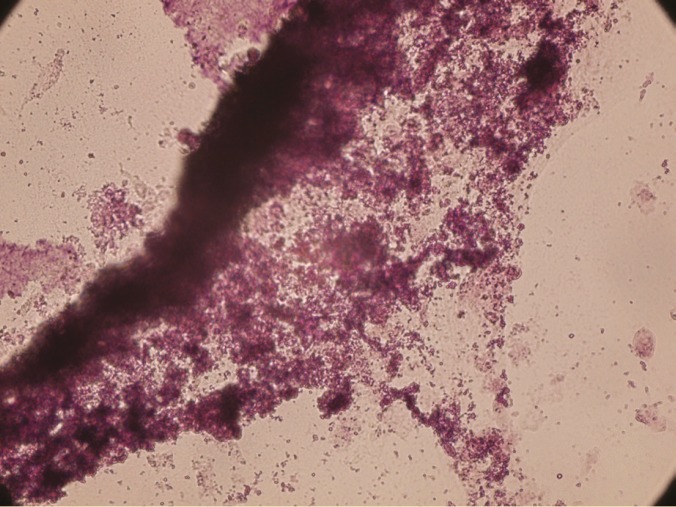
Deposition of calcium minerals with Alizarin Red (×100).

To investigate their capacity for mesodermal differentiation, the cells were induced to undergo adipogenic and osteogenic differentiation. The accumulation of lipid vacuoles was demonstrated by Oil Red O staining (Fig 5). Calcium deposition was revealed with Alizarin Red (Fig 6).

## DISCUSSION

Bone marrow MSCs (BM-MSCs) have been considered a good candidate for cell therapy. Several limitations exist for clinical application of BM-MSCs; the bone marrow aspiration was an invasive procedure and the proliferation and differentiation capacity of the cells decreased with increasing age. Therefore, fetal tissues, including umbilical cord blood, amniotic fluid, and placental tissue, have been considered alternative sources of stem cells [9, 10]. Fetal-derived MSCs, were immunologically privileged and produced only minimal immune reactivity [11, 12]. These cells expressed nonclassical human leukocyte antigen G (HLA-G), MHC class I antigens and express low levels of MHC class II antigens [2, 13, 14]. 

Additionally, the cells had normal karyotypes with no evidence of teratoma formation [2, 14]. 

Recent studies suggested that UC-MSCs do not require tissue matching; therefore, any donor can give cells to any person without rejection or need of immunosuppressive drugs [15]. In our study, AM-MSCs and WJ-MSCs had similar morphology, gene expression, and cell surface immunophenotype. The morphology of primary CP-MSCs and WJ-MSCs was similar to other MSCs, being fibroblastoid and spindle-like [16].

Previously reported protocols used enzymatic digestion of placental tissue, which was a costly and time-consuming process. Other disadvantages of enzymatic methods were use of bacterial-derived enzymes and potential contamination with endotoxins and xenoantigens [17, 18]. In this study, we have generated explant-derived MSCs from human umbilical cord and amniotic membrane, without the above-mentioned disadvantages.

According to Vellasamy, *et al*, [18] and Mareddy, *et al*, [19] findings, MSC cultures undergo senescence evidenced by slow growth and reduced differentiation potential.

In our study, the immunophenotype of the cells was unchanged, but continuous growth in culture and trypsinization may be a major cause of dead and loss of stemness.

Gene expression of our cells was similar to that of BM-MSC, ESC and other sources of MSC [20-22], which were essential to maintain their stemness. 

The amniotic membrane and umbilical cord is discarded as medical post-delivery waste. Therefore, MSCs from these easily accessible tissues, are promising cell population for tissue regeneration, immunomodulator in transplantation tolerance and autoimmunity.
